# Combatting Stigma Toward Individuals With Alzheimer's Disease and Related Disorders: The Role of Nostalgia

**DOI:** 10.1111/opn.70056

**Published:** 2025-12-13

**Authors:** Rhiannon N. Turner, Tim Wildschut, Constantine Sedikides

**Affiliations:** ^1^ Centre for Identity and Intergroup Relations, School of Psychology Queen's University Belfast Belfast Northern Ireland UK; ^2^ Centre for Research on Self and Identity, Psychology Department University of Southampton Southampton England UK

**Keywords:** Alzheimer's disease, approach behavioural tendencies, dementia, inclusion, nostalgia, outgroup attitudes, prejudice, stigma

## Abstract

**Introduction:**

There is limited evidence on how to tackle stigma toward individuals with Alzheimer's Disease and Related Disorders (ADRD). This study addressed this issue by examining how nostalgia (a sentimental longing for a personally experienced past) for experiences involving people with ADRD can be used to harness more positive attitudes and behavioral tendencies.

**Method:**

One hundred student nurses were randomly assigned to either recall a nostalgic or (in the control condition) an ordinary interaction involving a person with ADRD, before completing questionnaire measures of inclusion of people with ADRD (i.e., an outgroup) in the self (IOGS), stigma toward those with ADRD, and tendency to approach those with ADRD.

**Results:**

We found that experimentally induced nostalgia led to higher inclusion of people with ADRD in the self (IOGS; *F* [1, 98] = 5.69, *p* = 0.02, *η* ^2^ = 0.06) which was in turn linked to reduced stigma toward people with ADRD (*F* [, 98] = 8.07, *p* = 0.005, *η* ^2^ = 0.08) and a greater tendency to approach them (*F* [1, 98] = 4.35, *p* = 0.04, *η* ^2^ = 0.06).

**Conclusion:**

Induced nostalgia can reduce stigma and promote approach behavioral tendencies toward people within a medical context by promoting inclusion of people with ADRD in the self. These findings have interventional potential in a context where negative perceptions of people with ADRD can be problematic.

**Summary**

- What does this research add to existing knowledge in gerontology?
  ○ Among a sample of student nurses, experimentally induced nostalgia resulted in more positive attitudes and approach behavioural tendencies toward people with Alzheimer's Disease and Related Disorders (ADRD).
  ○ The effect of induced nostalgia on attitudes and approach behavioural tendencies toward people with ADRD was explained by higher inclusion of the entire group of people with ADRD (i.e., an outgroup) in the self (IOGS).
- What are the implications of this new knowledge for nursing care for and with older adults?
  ○ Stigma has been shown to have a detrimental effect on the treatment of patients by healthcare professionals,
  ○ Factors such as nostalgia and IOGS that ameliorate dementia stigma may help to combat such stigma in the context of nursing care with older people.
- How could the findings be used to influence practice, education, research, and policy?
  ○ Given that people regularly engage in nostalgia, the emotion has the potential to be used as a simple and practical tool among student nurses and nursing staff for reducing stigma toward individuals with ADRD.

## 1 Introduction

### 1.1 Stigma and Alzheimer's Disease and Related Disorders

Stigma refers to the reaction that the general population has toward people based on a negatively perceived group membership, and is characterised by adverse beliefs (stereotypes), emotional reactions (prejudice), and behaviours (discrimination) toward members of that group (Corrigan and Watson 2002). Stigma toward older adults (Butler 1969), for example, is marked by beliefs that, despite being friendly and good‐natured (Cuddy et al. 2005), older adults are forgetful, weak, ugly, and useless (Cuddy et al. 2005; Fineman 2014; North and Fiske 2012). In terms of prejudice, older adults commonly have to cope with disparaging and contemptuous attitudes (Kearney et al. 2000; Kite et al. 2005; Wilkinson and Ferraro 2002). Finally, in terms of discrimination, older adults are often overlooked professionally and medically: They are faced with work dismissals, are at risk for neglect and exploitation, and are denied essential medical interventions, compared to their younger counterparts (Bowling 1999; McCann and Giles 2002; Nelson 2005). Such treatment of older adults has aversive emotional, social, physical, and material consequences (Levy et al. 2009; Love and Torrence 1989; North and Fiske 2013).

An even more challenging case of stigma is evident toward people with Alzheimer's Disease and Related Disorders (ADRD). ADRD is associated with cognitive and emotional changes, including poor memory and autobiographical memory, a diminished sense of self and autonoetic consciousness (i.e., mental transportation), as well as increased aggression (El Haj et al. 2015; Greene et al. 1995; Link et al. 1999). People with ADRD face a double jeopardy in terms of prejudice: being older and bearing the stigma of cognitive impairment (Rodeheaver and Datan 1988). Symptoms such as aggression may mean that they are not imbued with the more positive traits (e.g., good‐natured) associated with older adults more generally (Burgener et al. 2015; Link et al. 1999). Indeed, recent research highlighted negative stereotypes toward those with dementia such as ‘violent,’ ‘angry all the time,’ ‘needy,’ and ‘crazy’ (Brookman et al. 2025). As such, they are at great risk of stereotyping, prejudice, and discrimination (Chan and Chan 2009).

This risk is reflected in the personal accounts of people with ADRD, which include experiences of social rejection, embarrassment, and changes to family dynamics (e.g., overemphasis on symptoms and inability, dismissal of retained abilities). These experiences contribute to attempts to hide the symptoms and are associated with reduced cognitive functioning and considerable psychological impairment (e.g., higher depression and anxiety, lower self‐esteem; Burgener et al. 2015). This risk is also reflected in the treatment of people with ADRD by health professionals, with nurses being a case in point (Chan and Chan 2009). Here, negative stereotypes and prejudicial attitudes may impede the quality of care provided, leading to discrimination (Broadaty et al. 2003). For example, nurses, relative to health‐care assistants, are more likely to view people with ADRD primarily through the lens of physical or mental dependence rather than through their unique personalities (Cooper and Coleman 2001).

### 1.2 Tackling ADRD Stigma

Given the negative social and health consequences of prejudice and discrimination against people with ADRD stigma, the development of interventions to tackle it is essential. Yet, evidence‐based approaches to this issue are sparse (Hermann et al. 2018). Extant interventions have primarily relied on exposing participants to written information about counter‐stereotypical individuals (van Gorp et al. 2012) with dementia. However, information‐based interventions are generally less effective than those that engage participants through direct experience and elicit affective responses toward the target group (e.g., Turner et al. 2007). Arguably, the most effective stigma‐reduction intervention across a broad array of groups is positive intergroup contact (Allport 1954; Pettigrew and Tropp 2006; van Assche et al. 2023). However, to our knowledge, such an intervention has been the basis for only one qualitative study (Harris and Caporella 2014). The results indicated that participation in an intergenerational choir of university students, individuals with dementia, and their family members could reduce social discomfort and promote positive attitudes. Although promising, this type of intervention is time‐consuming and may not be practical in a busy healthcare setting. Moreover, none of the evaluations of ADRD stigma reduction to date have used a comparison control group, nor have they involved individuals in the healthcare domain.

In light of the paucity of research on such interventions and the limitations identified, it is important to explore alternative approaches to addressing ADRD stigma. Interventions that target health professionals, and in particular student nurses, are needed. Such interventions may assist student nurses in internalizing positive attitudes toward people with ADRD, thus promoting career‐long anti‐discriminatory practices (Chan and Chan 2009). We consider the construct of nostalgia as a potential intervention.

### 1.3 Nostalgia as a Promising Intervention Platform

Nostalgia is a social emotion involving the recall of significant autobiographical events, often involving close others, such as family and friends (Sedikides et al. 2015; Sedikides and Wildschut 2016; Wildschut et al. 2010). It is experienced frequently (i.e., several times a week; Sedikides et al. 2015), virtually by everyone (Boym 2001), and across cultures (Hepper et al. 2014). It is bittersweet in its content, although more sweet (positive: contentment, happiness) than bitter (negative: missing, longing; Sedikides et al. 2015; Sedikides and Wildschut 2016; Wildschut et al. 2006), and is self‐relevant, arising from fond and meaningful memories involving one's personal past (Sedikides and Wildschut 2016; van Tilburg et al. 2018). Nostalgia strengthens sociality, promoting feelings of being protected, supported, and loved (Abeyta et al. 2015; van Tilburg et al. 2018; Zhou et al. 2008).

We capitalised on nostalgia to design an intervention experiment. In particular, we experimentally induced nostalgia with a validated procedure (Gravani et al. 2018; Turner and Stathi 2023; Turner et al. 2012; Turner et al. 2013; Turner et al. 2018). We instructed student nurses in the nostalgia condition to bring to mind, and nostalgize about, a social exchange with person with ADRD. We instructed student nurses in the control condition to bring to mind and reflect upon an ordinary social exchange with a person with ADRD.

We assumed that participants in the nostalgia condition, in experiencing greater social connectedness, would feel closer to the person with ADRD than control participants. That is, they would feel as if they included the other person in their self‐concept (Aron et al. 1992). Hence, we asked all participants to indicate the extent to which they included in the self the entire group “people with ADRD” (i.e., the outgroup). Interpersonal closeness entails becoming part of each other's identity and perspectives (Tropp and Wright 2001). Provided group membership is salient (i.e., the remembered person with ADRD is seen as belonging to the ADRD group), not only the individual, but the entire outgroup will be incorporated in the self (Sassenberg and Matschke 2010).

Taken together, we reasoned that, if experimentally induced nostalgia strengthens the mental bond between self and other (i.e., a person with ADRD), then it will strengthen the mental bond between the self and people with ADRD. The outgroup will now become part of who one is (i.e., one's self‐concept). Further, and crucially, given that individuals have positive attitudes toward themselves (Sedikides and Gregg 2008) and will extend these attitudes to the other (i.e., a person with ADRD), they will by implication also adopt more positive (i.e., less prejudicial) attitudes and a greater tendency to approach (e.g., get to know, find out more about) people with ADRD (Aron et al. 1991).

In summary, the significance of this work lies in experimentally demonstrating an effective intervention to ADRD among student nurses and highlighting the processes by which the intervention works. Given the implications for how those with ADRD are treated in medical settings, there are potential benefits for staff training and for the well‐being of patients in gerontology settings.

### 1.4 Hypotheses

We hypothesize that nostalgic (relative to control) participants will include “people with ADRD” in the self. In turn, inclusion of the outgroup in the self will be associated with improved attitudes and a greater tendency to approach people with ADRD; statistically speaking, we expect inclusion of the outgroup in the self to mediate the beneficial effect of nostalgia on attitudes and approach tendencies toward people with ADRD.

## 2 Materials and Methods

### 2.1 Participants and Design

We tested 100 undergraduate student nurses in Southampton, UK (85 women, 15 men), aged between 18 and 51 years (*M* = 23.94, SD = 7.47). All participants provided informed consent. We did not screen participants. Participants were recruited and completed the study in a lecture theatre prior to a lecture, and completed the study voluntarily. We used a between‐subjects design, randomly assigning participants to the nostalgia or control condition using a random‐number table. No significant differences were observed in terms of gender (*F* (1, 98) = 0.42, *p* = 0.52, *η* ^2^ = 0.004) or age (*F* (1, 98) = 2.22, *p* = 0.14, *η* ^2^ = 0.02) between the two conditions.

### 2.2 Procedure and Measures

We induced nostalgia with a validated manipulation (Gravani et al. 2018; Turner and Stathi 2023; Turner et al. 2012; Turner et al. 2013; Turner et al. 2018) after adjusting it to refer to a person with ADRD. Participants in the nostalgia condition read a dictionary definition of nostalgia (“sentimental longing of wistful affection for the past”) and were instructed:

Recall a nostalgic event in your life that involved interacting with a person you know who has Alzheimer's disease or related disorder. We would like you to choose someone you know well. This could be a (present or former) acquaintance, patient, friend, partner or family member. Please write down the initials of this person with Alzheimer's disease or related disorder. Please think of a past event involving this person that makes you feel most nostalgic. Bring this nostalgic experience to mind. Immerse yourself in the nostalgic experience involving this person, remembering what it was like and how you felt at the time you interacted with this person. How does it make you feel?

Participants in the control condition were instructed:

Recall an ordinary event in your life that involved interacting with a person you know who has Alzheimer's disease or related disorder. We would like you to choose someone you know well. This could be an (present or former) acquaintance, patient, friend, partner or family member. Please write down the initials of this person with Alzheimer's disease or related disorder. Please think of a past event involving this person that was normal or every day. It could be something like a normal visit to their house, or doing a chore for someone. Bring to mind an objective record of this event and think it through as though you were a scientist or historian recording factual details. Now we would like you to spend a few minutes imagining that you are back at this event.

After spending 5 min on this task, participants completed the following measures in the specified order.

#### 2.2.1 Manipulation Check

Participants listed four keywords relevant to the event. To examine whether the manipulation induced nostalgia, we content‐analysed these keywords with the Linguistic Inquiry and Word Count (LIWC) software program (Pennebaker et al. 2007). Specifically, we calculated the percentage of nostalgia words captured by the wordlist in the Nostalgia Dictionary (e.g., bittersweet, comfort, familiar), which has been validated, including by establishing convergence with independent raters (Chen et al. 2023).

#### 2.2.2 Inclusion of the Outgroup in the Self

Participants were presented with seven Venn diagrams (i.e., pairs of circles; Aron et al. 1992). For each diagram, the left circle represented the participant, whereas the right circle represented “People with Alzheimer's Disease and Related Disorders.” The pairs ranged from non‐overlapping to progressively overlapping to almost completely overlapping (1–7). Thus, lower numbers reflected no or little inclusion of the outgroup in the self, whereas higher numbers reflected stronger inclusion of the outgroup in the self. The specific instructions stated:

Now please think about your relationship with people with Alzheimer's disease or related disorder in general. Imagine that one circle represents you and one represents *all* people with Alzheimer's disease or related disorder. Keeping in mind the nostalgic event you just recalled, describe how close you feel to people with Alzheimer's disease or related disorder by circling the picture which best describes your relationship with them.

#### 2.2.3 Attitudes Toward People With Alzheimer's Disease and Related Disorders

Attitudes have affective, cognitive, and behavioural components (Eagly and Chaiken 1993), which are captured by the 20‐item Dementia Attitude Scale (O'Connor and McFadden 2010). Sample items are “I am afraid of people with ADRD” (affective component), “People with ADRD can be creative” (cognitive component), and “I cannot imagine caring for someone with ADRD” (behavioural component). After reverse‐scoring 10 items, we computed an aggregate score (1 = *strongly disagree*, 6 = *strongly agree*; *α* = 0.86). Higher scores indicate a more positive attitude toward people with ADRD.

#### 2.2.4 Approach Behavioural Intentions Toward People With Alzheimer's Disease and Related Disorders

Participants were asked: ‘With this nostalgic event in mind, when thinking about people with Alzheimer's disease or related disorder…’. They then responded on three items: ‘I want to talk to them’, ‘I want to find out more about them’ and ‘I want to spend time with them’ (1 = *not at all*, 6 = *very much*; α = 0.89; Mackie et al. 2000). Higher scores indicate greater intention to approach people with ADRD.

## 3 Results

### 3.1 Manipulation Check

To examine whether the manipulation induced nostalgia, we content‐analysed participants' keywords with the Linguistic Inquiry and Word Count software program (Pennebaker et al. 2007). We used the program to calculate the percentage of nostalgia words captured by the wordlist in the validated Nostalgia Dictionary (Chen et al. 2023; e.g., bittersweet, comfort, familiar). Participants in the nostalgia condition had a higher nostalgia score (*M* = 32.69, SD = 24.88) than those in the control condition (*M* = 15.11, SD = 20.66), *F* (1, 94) = 14.20, *p* < 0.001, *ƞ* ^2^ = 0.13. Our manipulation was effective.

### 3.2 Inclusion of the Outgroup in the Self

**FIGURE 1:**
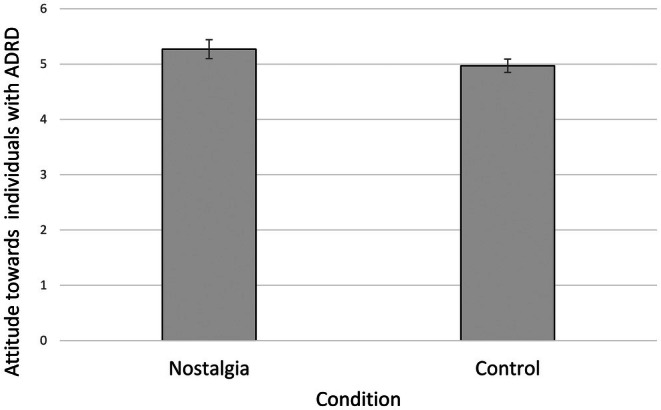
Effect of nostalgia on attitudes toward people with ADRD, with 95% CI error bars.

As hypothesized, nostalgic participants (*M* = 4.49, SD = 1.52) reported higher IOGS than controls (*M* = 3.76, SD = 1.48), *F* (1, 98) = 5.69, *p* = 0.02, *ƞ* ^2^ = 0.06 (see Figure 1).

### 3.3 Attitudes Toward People With Alzheimer's Disease and Related Disorders

**FIGURE 2:**
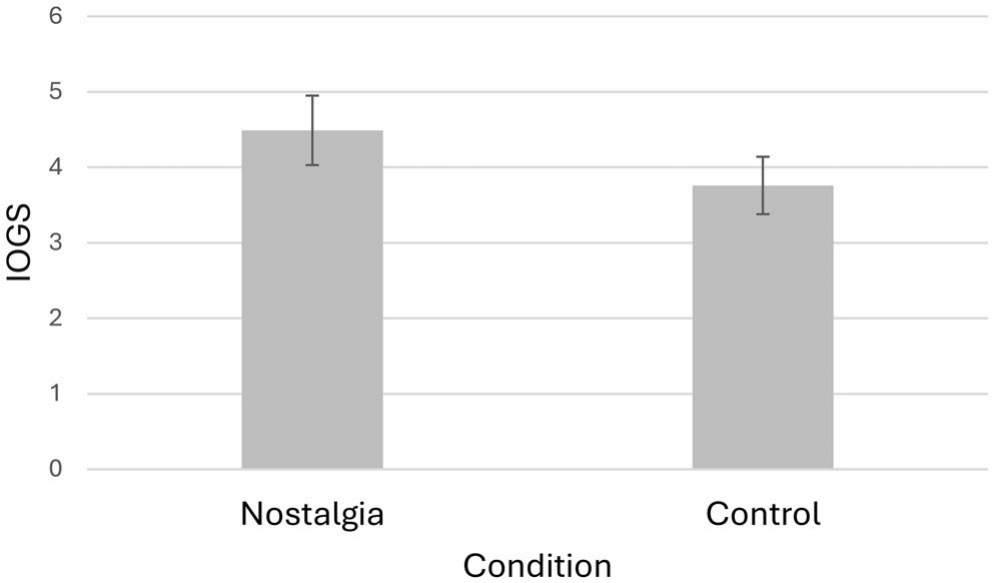
Effect of nostalgia on IOGS toward people with ADRD, with 95% CI error bars.

As hypothesized, nostalgic participants (*M* = 5.27, SD = 0.59) expressed more positive attitudes toward people with ADRD than controls (*M* = 4.97, SD = 0.49), *F* (1, 98) = 8.07, *p* = 0.005, *ƞ* ^2^ = 0.08 (see Figure 2). We ran a moderated regression with the interaction term of nostalgia and IOGS to examine whether this influenced attitudes over and above the main effects of each variable, but found no evidence that the impact of nostalgia was moderated by participants' IOGS score (R Squared Change = 0.01, *F* Change = 1.7, *p* = 0.30).

### 3.4 Approach Behavioural Tendencies Toward People With Alzheimer's Disease and Related Disorders

**FIGURE 3:**
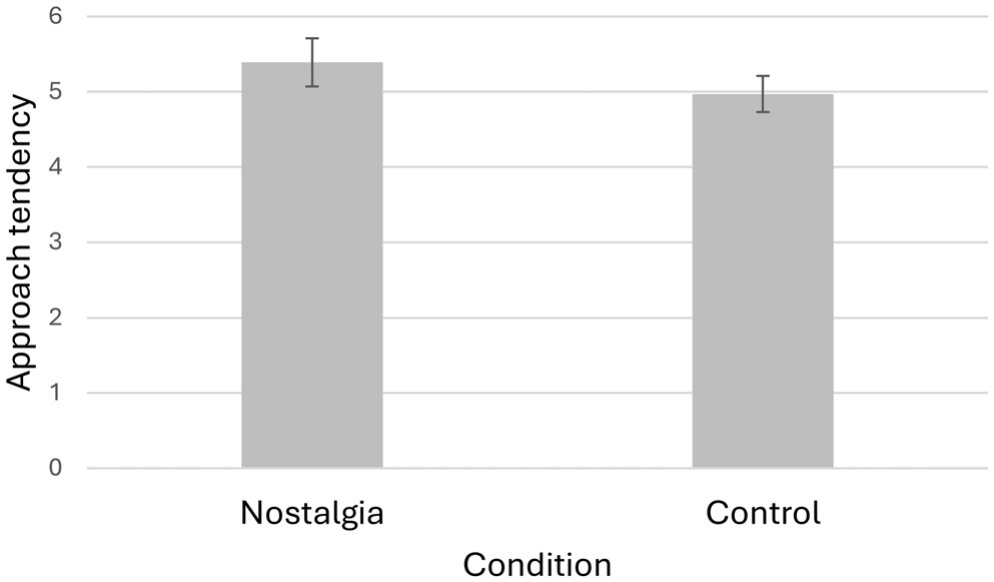
Effect of nostalgia on approach behavioral tendencies toward people with ADRD, with 95% CI error bars.

As hypothesized, nostalgic participants (*M* = 5.39, SD = 0.90) expressed a greater tendency to approach people with ADRD than controls (*M* = 4.97, SD = 0.83), *F* (1, 98) = 4.35, *p* = 0.04, *ƞ* ^2^ = 0.06 (see Figure 3). We ran a moderated regression with the interaction term of nostalgia and IOGS to examine whether this influenced attitudes over and above the main effects of each variable, but found no evidence that the impact of nostalgia was moderated by participants' IOGS score (R Squared Change = 0.03, *F* Change = 2.89, *p* = 0.09).

### 3.5 Mediational Analyses

Descriptive statistics and correlations between the study variables are shown in Table 1.

**TABLE 1:** Descriptive statistics and correlations for study variables.

Variable	M	SD	1	2	3	4
1. Condition (control = −1, nostalgia = 1)	—	—	—			
2. IOGS	4.06	1.53	0.23*	—		
3. Attitude toward people with ADRD	5.09	0.54	0.28**	0.40***	—	
4. Approach tendency toward people with ADRD	5.14	0.88	0.24*	0.23**	0.65***	—

* *p* < 0.05.

** *p* < 0.01.

*** *p* < 0.001.

As indicated previously, nostalgic participants reported greater IOGS than control participants, *β* = 0.73, SE = 0.30, *t* (98) = 2.39, *p* = 0.02. Controlling for the nostalgia manipulation, IOGS and attitudes toward people with ADRD were positively related, *β* = 0.12, SE = 0.03, *t* (98) = 3.72, *p* < 0.001. When IOGS was controlled for, the effect of nostalgia on attitudes toward people with ADRD remained significant, albeit reduced, *β* = 0.21, SE = 0.10, *t* (98) = 2.07, *p* = 0.04. Controlling for the nostalgia manipulation, IOGS and approach tendencies toward people with ADRD were also positively related, *β* = 0.28, SE = 0.07, *t* (73) = 2.53, *p* = 0.02. When IOGS was controlled for, the effect of nostalgia on approach tendencies was no longer significant, *β* = 0.17, SE = 0.19, *t* (73) = 1.50, *p* = 0.14.

We used PROCESS (Hayes 2013; Model 4; 5000 bootstrap samples) to test the indirect effects (denoted as *ab*) of nostalgia on each outcome via IOGS. This indirect effect of nostalgia on attitudes toward people with ADRD was significant, *ab* = 0.0885, SE = 0.0480, 95% CI = [0.0162, 0.2046], as was the indirect effect of nostalgia on approach tendencies toward people with ADRD, *ab* = 0.1187, SE = 0.0750, 95% CI = [0.0008, 0.2948]. In all, IOGS mediated the effect of nostalgia on attitudes and behavioural tendencies toward people with ADRD.

## 4 Discussion

Universal ageing trends toward a sharp rise in the prevalence of ADRD (World Health Organization 2011), coupled with a double jeopardy regarding prejudice that is experienced by ADRD patients (Brookman et al. 2025; Hermann et al. 2018), means that it is of utmost importance to develop practical yet effective approaches to tackle stigma against people with ADRD. In this study, we examined the impact of a nostalgia‐based intervention in promoting more positive perceptions of people with ADRD among student nurses.

We hypothesized that reflecting nostalgically on a past interaction with an individual with ADRD would be associated with improvements in attitudes and a tendency to approach people with ADRD. We also hypothesized that this effect would be mediated by stronger IOGS. Results confirmed these hypotheses, revealing that recollecting nostalgically about an individual with ADRD was associated with strengthened IOGS. In turn, this increased IOGS was associated with improved attitude and approach tendencies toward people with ADRD. Although our mediational analysis cannot imply causality (Fiedler et al. 2011), given the significance of each pathway, our results remain informative. Our results confirm and extend previous literature on nostalgia as a prejudice reduction technique and therefore have encouraging implications for future research.

### 4.1 Implications

Our findings confirm the effectiveness of nostalgia as a potential intervention to curtail prejudice against individuals with ADRD. We support the proposition that nostalgia's recently recognised sociality function provides a basis for strengthened IOGS (Tropp and Wright 2001). As individuals reflect nostalgically on a past encounter with an identified person with ADRD, they feel closer to them and include them in their own identity (Sassenberg and Matschke 2010). Given the distinctiveness of ADRD group membership, IOGS is in turn transferred to the entire outgroup, resulting in inclusion of the entire outgroup in the self (Brown and Hewstone 2005). This IOGS results in the individual relating to the outgroup, treating the outgroup like the self and therefore, improving attitudes held toward them.

Previously examined interventions have several limitations. Some may be too costly, time‐consuming, or impractical in healthcare settings (e.g., intergroup contact; Harris and Caporella 2014). Others, which draw on counter‐stereotypical descriptions of individuals with ADRD (e.g., van Gorp et al. 2012) have promise, but may be less effective than those that draw on actual experience and elicit affective responses to the target group (Turner et al. 2007). Given that nostalgia is experienced universally and effortlessly, with people reporting nostalgic experiences about three times a week (Sedikides et al. 2015), we argue that it offers a more concrete, spontaneous, and vivid approach to prejudice reduction than vignette‐based approaches. Such an experience also has some advantages over intergroup contact interventions, which require time and effort. Moreover, it is likely to benefit those with limited opportunities for direct contact involving individuals with ADRD. We conclude that nostalgia has the potential to provide an effective solution to changing the costly and hardwired negative stereotypes held toward individuals with ADRD. Indeed, this brief nostalgia intervention produced more positive perceptions of people with ADRD despite this being an especially challenging form of stigma, due to the double jeopardy to prejudice faced by these individuals who are both older and experiencing mental/cognitive impairment (Hermann et al. 2018). This is a testament to the strength of the approach. Future studies should examine the impact of incorporating nostalgia exercises as part of nursing and medical students' classroom activities when learning about ADRD, and during continued professional development for qualified nurses and doctors.

### 4.2 Limitations

Our findings, relating to a relatively small student sample of nurses, cannot be generalised to certified health professionals. Some researchers highlight that students may be more open to intergroup representations than the general population (Smeekes and Verkuyten 2015). We must therefore consider that the social functions of nostalgia that are associated with prejudice reduction, such as IOGS, may not be as beneficial in non‐student samples. Converging research examining such prejudice from the perspective of nursing or care staff is required to affirm nostalgia's status as an effective technique for reducing prejudice against individuals with ADRD.

The age of our sample must also be considered. Our sample of nursing students had a mean age of 23. As recent evidence suggests that younger adults are less likely to report negative and derogatory stereotypes regarding individuals with dementia in the first place, and are also more likely to show an association between dementia knowledge and reduced stigma (Brookman et al. 2025), further research is required to ascertain the impact of nostalgia on more established (and therefore potentially older) individuals working in healthcare, for example qualified nurses across ages and career stages. It is also notable that our sample comprised a majority of women, as is common in nursing, making it difficult to test gender differences. Future research with a larger sample could address this concern.

Our research only considered participant attitudes at one point in time. Future research should test whether the immediate attitude change is maintained in the long term. Whereas a one‐off approach may not result in long‐term attitude change, given the brevity of the intervention tested here, it may be practical to repeat it at regular intervals in the workplace to ensure lasting change. Further investigations are necessary to examine the efficacy of this approach. Furthermore, future work should address the question of how behavioural intentions toward outgroups relate to actual performed behaviour in the future. Such work may explore the effects of nostalgia as a prejudice reduction intervention either individually or when implemented alongside other established prejudice reduction techniques. Although implementing nostalgia as an intervention in itself would eliminate the need for new, actual contact with an individual with ADRD, integrating it alongside intergroup contact may strengthen the desired effects. Finally, we acknowledge that there may be more than one pathway leading to our findings; another factor we did not test may mediate the relationship between nostalgia and improved attitudes toward individuals with ADRD. Indeed, research examining the relation between nostalgia and stigma toward other groups has identified mediating mechanisms such as social connectedness, intergroup trust, and lowered intergroup anxiety (e.g., Turner and Stathi 2023; Turner et al. 2012; Turner et al. 2013; Turner et al. 2018). Exploring alternative pathways would be noteworthy in follow‐up investigations. Finally, we did not assess participants' personal experience with people with ADRD, or experience working in a dementia care setting. Given the strong established relation between contact and outgroup attitudes (e.g., van Assche et al. 2023), this would be a valuable inclusion in future research to examine whether personal contact experience influences intervention efficacy.

## 5 Conclusion

Nostalgia was effective at reducing prejudice toward people with ADRD among a sample of nursing students. Nostalgia's sociality implies that individuals reflecting nostalgically feel closer to ADRD patients and begin to include them in the self, improving their attitudes toward them. Given that the prevalence of ADRD is increasing, future research may draw on our findings to evaluate the long‐term efficacy of implementing nostalgia as a prejudice‐reduction intervention in healthcare settings, either alone or alongside other interventions as part of a multi‐pronged approach.

## Author Contributions

**Rhiannon N. Turner:** analysed data, led on writing of the paper and response to editor and reviewers. **Constantine Sedikides and Tim Wildschut:** designed study, collected data, provided feedback on drafts of the paper.

## Ethics Statement

The research reported in this data received ethical approval from the Research Ethics Committee in the School of Psychology, University of Southampton.

## Conflicts of Interest

The authors declare no conflicts of interest.

## Data Availability

The data that support the findings of this study are available on request from the corresponding author. The data are not publicly available due to ethical restrictions.
